# Screen Time and Executive Function in Toddlerhood: A Longitudinal Study

**DOI:** 10.3389/fpsyg.2020.570392

**Published:** 2020-10-22

**Authors:** Gabrielle McHarg, Andrew D. Ribner, Rory T. Devine, Claire Hughes

**Affiliations:** ^1^Centre for Family Research, University of Cambridge, Cambridge, United Kingdom; ^2^Learning Research and Development Center, University of Pittsburgh, Pittsburgh, PA, United States; ^3^Department of Psychology, University of Birmingham, Birmingham, United Kingdom

**Keywords:** screen time, executive function, inhibition, working memory, toddler, longitudinal

## Abstract

Technology is pervasive in homes with young children. Emerging evidence that electronic screen-based media use has adverse effects on executive functions may help explain negative relations between media use and early academic skills. However, longitudinal investigations are needed to test this idea. In a sample of 193 British toddlers tracked from age 2 to 3 years, we test concurrent and predictive relations between screen use and children’s executive function. We find no concurrent association between screen use and executive function; however, screen time at age 2 is negatively associated with the development of executive functions in toddlerhood from age 2 to 3, controlling for a range of covariates including verbal ability. Implications for parenting, education, and pediatric recommendations are discussed.

## Introduction

Parents around the globe have, since the advent of the television, been questioning the effects of screens on children’s development – leading to a “moral panic” surrounding children’s electronic screen-time (Drotner, 2013). This debate has, in part, been fueled by reports of negative consequences of screen time during childhood and adolescence. For example, screen viewing has been associated with reduced sleep in infancy and toddlerhood (e.g., Cheung et al., 2017; Ribner and McHarg, 2019) and in adolescence (e.g., Hisler et al., 2020; Magee and Blunden, 2020). In addition, increased screen time is associated with increased sedentary behavior and obesity (e.g., Robinson et al., 2017); and television has been negatively correlated with both parental engagement (e.g., Mendelsohn et al., 2008; Christakis et al., 2009; Kirkorian et al., 2009) and children’s language and literacy skills (Ribner et al., 2020). Each of these associations is important for parents and clinicians to consider when addressing questions about the potential risks of screen time. Understanding digital media’s impacts on cognition is therefore vital to support those who care for children. However, few studies have applied longitudinal designs to explore how screen time might affect toddlers’ cognitive development. Addressing this gap, the current study investigates variation in children’s executive functions at 36-months of age in relation to ratings of screen use gathered both concurrently and 12-months earlier (i.e., at 24-months).

Executive functions (EF) are a multidimensional set of skills comprised of inhibitory control, working memory, and cognitive flexibility. These skills are implicated in classroom behavior and learning, and in the pursuit of goal-directed cognitions, actions, and behavior more broadly (Diamond, 2013). EF and its component parts develop from very early childhood through early adulthood, with substantial individual differences in the pace of development (e.g., Diamond, 2013). While the factor structure of EF—particularly in infancy and toddlerhood—is unclear (e.g., Willoughby et al., 2010, 2012; Lerner and Lonigan, 2014; Miller and Marcovitch, 2015; Holmboe et al., 2018; Devine et al., 2019; Fiske and Holmboe, 2019), it is evident that individual differences in very early EF (regardless of how it is operationalized) are associated with children’s ability to regulate their behavior (Vernon-Feagans et al., 2016; Hughes et al., 2020), engage in goal-directed behavior (e.g., Hendry et al., 2016), understand others’ thoughts and feelings (Hughes et al., 2009; Devine and Hughes, 2014), and successfully transition to school settings (Hughes et al., 2009; Mulder et al., 2017; Willoughby et al., 2017).

Several factors portend individual differences in EF including neurological differences (e.g., Short et al., 2019), early attention (e.g., Blankenship et al., 2019; Devine et al., 2019), and cognitive training (Scionti et al., 2020). Furthermore, several environmental factors including aspects of parenting (e.g., Hughes et al., 2013; Fay-Stammbach et al., 2014; Hughes and Devine, 2019), child care (e.g., Duncan et al., 2019), and stress (Blair, 2010) are associated with the development of young children’s EF. While many of these factors might be outside parents’ locus of control, screen exposure may be a more controllable environmental factor related to EF development.

Prior research has established that increased screen time is typically associated with lower EF (e.g., Barr et al., 2010; Lillard and Peterson, 2011; Nathanson et al., 2014; Cliff et al., 2018); notably, this association is evident as early as infancy. Specifically, McHarg et al. (2020) used a propensity score matching approach and found that, all things being equal, having regular screen exposure of any amount at 4 months was related to worse inhibitory control, though there was no association of screen exposure with working memory or cognitive flexibility. However, it is important to note that screen exposure in infancy is fundamentally different than later screen exposure. Infants do not begin to process information presented on screens for more than 3–5 s (for summary, see Kirkorian et al., 2017), and young children do not begin to understand even child-directed content until age 2 (Anderson and Subrahmanyam, 2017; Hipp et al., 2017), suggesting that all screen time in infancy might be effectively treated as adult-directed content and/or background media.

Importantly, though, longitudinal associations between screen time and EF appear to extend beyond infancy. One study showed that viewing less television and less overall media exposure at age 2-years were related to higher self-regulation at 4-years (Cliff et al., 2018). Another found that higher levels of exposure to non-child-directed screen content at 12 to 14 months of age were related to lower inhibitory self-control and metacognition skills at age four (Barr et al., 2010). Experimental findings bolster these correlational results. Lillard and Peterson (2011) found that 4-year-old children who watched a fast-paced cartoon, rather than either an educational cartoon or no television, performed significantly worse on EF tasks immediately after watching. Separately, Huber et al. (2018) found that children were less likely to delay gratification after viewing a cartoon than after playing an educational app. Collectively, these findings suggest temporary “state” effects on EF—that is, effects that are short lasting and might be associated with a third variable such as mood or attention and will fade out over a brief period—but say nothing about effects on individual differences in chronic or lasting “traits”—that is, effects that are longer lasting that might have negative downstream consequences.

For parents, educators, and clinicians, “screen time” is a loaded term and may reflect a number of different definitions for parents and researchers. Technological advance and the growing availability of mobile technologies far outpace research, making it difficult to know what is “right” for children, particularly in an era when screens are increasingly used for educational purposes. In addition, large immobile screens that pervade the everyday landscape (e.g., on the street, in shop windows, in restaurants) and mobile devices are fundamentally different, as one can be carried around and used on buses and trains and one must be watched from one location, adding to the difficulty of making recommendations. Indeed, mobile devices offer opportunities for interactive use (e.g., playing games or taking photographs) that offer opportunities to practice working memory, planning and inhibition, and so may even improve executive functions (e.g., Huber et al., 2018).

Many international health organizations (e.g., the American Academy of Pediatrics, as stated in Chassiakos et al., 2016; World Health Organization, 2019) recommend a daily limit of less than 1 h of screen time for children between the ages of 2 and 4. Importantly—in part due to the correlational nature of most extant research and the rapidly evolving landscape of technology—the consequences of extended screen viewing are still unclear, inconclusive, and misunderstood, leading some health organizations (e.g., Royal College of Pediatrics and Child Health, as stated in Viner et al., 2019) to avoid making any recommendation about screen viewing limits for young children.

The present study contributes to two major gaps in the literature. First, the majority of extant research has used cross-sectional data, making it impossible to disentangle directionality in the relations between media use and EF. Those few studies that have investigated longitudinal associations have found that screen time predicts worse EF—or at least components thereof—at later time points (e.g., McHarg et al., 2020). However, it is also important to note that much of the work examining relations between media use and EF has been limited to either infants (e.g., McHarg et al., 2020) or preschool-aged children (e.g., Barr et al., 2010). There has been little exploration of these relations in toddlerhood, particularly in recent years when screen time has become increasingly mobile. Filling in this developmental gap is key for understanding how executive function develops, especially as digital media use increases with age (e.g., Madigan et al., 2019). Though executive function may begin to develop in infancy (e.g., Hughes et al., 2020), development does not stop until adulthood (e.g., Friedman et al., 2016) and toddlerhood may be a key period for establishing EF skills. As such, in the first study of its kind, our second aim is to extend prior investigations to better understand consequences of extended screen use in toddlerhood using a large prospective longitudinal study in the United Kingdom. We expect that increased digital media use will be associated with lower executive function, both concurrently and longitudinally, at 36-months of age.

## Materials and Methods

### Participants

Participants were recruited as a part of a larger longitudinal study of parents and their first-born children. To be eligible for the current study, potential participants had to: (1) be first-time parents, (2) be expecting to deliver a healthy singleton baby, (3) be planning to speak the English as the child’s primary language, and (4) have no history of severe mental illness (e.g., psychosis) or substance misuse. We recruited 213 couples expecting their first child attending prenatal classes and appointments at local hospitals in the East of England. All parents were co-habiting, first-time parents planning to speak English as a primary language with their child. All remaining participants were born full term (after 36 weeks) and without birth complications. Of families recruited, 194 families agreed to participate in a home visit when their children were 24 months old and 170 children were visited when they were 36 months old. Families who completed all data collection when children were 24 months of age (*n* = 179) were included in analyses; participants who completed data collection at 36 months of age but not 24 months (*n* = 7) were excluded. Participants who completed data collection at 24 months but not 36 months of age were included in all inferential analyses and missing data were accounted for using Full Information Maximum Likelihood estimation; however, sample sizes for descriptive analyses vary as the number of participants who completed each task or for whom parents provided data may differ from one task to another.

All procedures performed were in accordance with the Ethical standards of the Institutional and/or National Research Committees involved and were acceptable according to the 1964 Helsinki declaration and its later amendments or comparable Ethical standards. The National Health Service (NHS, United Kingdom) Research Ethics Committee approved the study protocol (REF: 14/LO/1113).

### Procedure

Data collection occurred at five timepoints: the third trimester of pregnancy, and when the children were 4, 14, 24, and 36 months of age; for the present investigation however, only data from the 24-, and 36-month timepoints are used. Data collection took place in children’s homes when children were 24 months of age [T1, *M*_age_(179) = 24.29 months, *SD* = 0.85]; when children were 36 months of age [T2, *M*_age_(163) = 36.24 months, *SD* = 1.09], 109 children were seen at their nursery, 56 children were seen at home, and five children were seen with their childminder/nanny at the childminder’s house. All protocols were administered in a standardized order by trained graduate students or postdoctoral researchers.

### Measures

#### Screen Exposure

Both mothers and fathers completed separate similar online questionnaires which included questions about their children’s technology use when their children were approximately 24-months old and 36-months old. Parents reported the amount of time children watched television and used other technology (e.g., touchscreens and computers) on weekdays and weekends in response to an item that asked “Thinking about your child how much time does your child spend doing each of the following activities at home on a typical WEEKDAY/WEEKEND DAY.” Parents responded to four items, one each asked the amount of time their child spent engaging with TV or DVDs, computers, books, and touch screen devices (e.g., tablet, phone). Each item was rated on the same scale in which parents were asked to respond with how much time (choosing from “not used”, “less than or equal to 30 min,” “30 min to an hour,” “1 to 2 h,” “3 to 4 h,” or “greater than or equal to 5 h”) their child spent engaging with TV or DVDs, computers, books, and touch screen devices per day. These answers were transformed to the numerical value in the middle of the range in minutes (i.e., corresponding to the above options, transformed values were 0, 15, 45, 90, 330, and 600 min) and values for screen-based devices were summed. Time spent engaging with books was not included in the constructed variables because, though it is considered a type of media, it was impossible to disaggregate whether parents were reporting on paper or digital books; in addition, book sharing in both physical and electronic forms is fundamentally different from viewing media (e.g., Lillard et al., 2015; Ribner et al., 2020).

Wherever possible, an average of mother- and father-reported child media use was used as the variable of interest; if only one parent responded, that parent’s values were used. I total, there were data available for *n* = 179 children at T1 and for *n* = 149 children at T2. At T1, 170 mothers and 171 fathers completed questionnaires; at T2, 145 mothers and 128 fathers completed questionnaires. Mother and father reports on the resulting aggregates comprising screen time on TV/DVD, computers, and touchscreen devices were quite similar [T1, *r*(160) = 0.471, *p* < 0.001; T2, *r*(122) = 0.628, *p* < 0.001].

Importantly, for the current project, screen time was considered to be non-interactive and mostly involved viewing television content (as opposed to being interactive and contingent). Parent interviews completed with families from this sample (180 mothers and 179 fathers) for a separate study revealed that mobile device screen usage, in addition to television viewing, in toddlerhood mainly involved children viewing television content in the vast majority of cases—most children were not using applications. Therefore, a decision was taken to combine parents’ answers to questions about all devices (i.e., TV or DVDs, computers, touchscreen devices) into one screen time variable by averaging values for each that is understood to be mostly non-interactive.

#### Executive Function

Executive function was measured using a series of direct assessment tasks administered in a standardized order. Administration of tasks and scoring at each time point are detailed below. At T1, children completed three EF tasks: A multi-location search task, followed an A-not-B-style shifting task, then a Stroop task; this battery of assessments is described in greater detail elsewhere (Hughes et al., 2020) and is reviewed below. At T2 months, children completed four EF tasks: A multi-location search task, a Dimensional Change Card Sorting task, a Stroop task, and a self-ordered pointing task. Scoring for T1 was the same as prior investigations using this same assessment battery (i.e., Hughes et al., 2020); scoring conventions for T2 was designed to be as similar to T1 as possible while still maintaining an adequate distribution of scores and providing equal weight in the resulting aggregate score to each assessment. Assessment took approximately 10 min at each time point.

##### EF at 24 months

Children first completed a Multi-Location Search Task as a measure of working memory (Miller and Marcovitch, 2015). Children searched for five cars hidden in five toy garages (one in each garage) that were distinct in both color and size after a delay of 5 s between each search. The task continued until the child retrieved all cars or made three consecutive errors. Children passed, and thus received a score of “1,” if they retrieved all of the hidden cars (*n* = 107). If they did not find all five cars, they received a score of “0” (*n* = 75).

Children then completed the Ball Run Task (Devine et al., 2019) as a measure of cognitive flexibility. In the learning phase, the examiner demonstrated how to activate a musical switch by placing a colored ball (e.g., red) into one of two colored holes (e.g., red hole). The other hole (e.g., green) was sealed from beneath and could not be used to activate the switch. Children completed 6 learning trials with feedback. In the reversal phase, the examiner demonstrated how to activate the toy by placing a different colored ball (e.g., green) into the previously unused hole (e.g., green); the color of the ball always matched the color of its intended hole in an effort to test cognitive flexibility rather than inhibition. The original hole was sealed from beneath and could no longer be used to activate the switch. Children completed 6 reversal trials with feedback. Children passed a phase if they performed correctly on 4 or more trials, such that they could receive a score of “0” (*n* = 30) if they passed neither the learning nor reversal phase; “1” (*n* = 66) if they passed only the learning phase; or “2” (*n* = 89) if they passed both the learning and reversal phase. Order of administration (red vs. green hole and ball first) was counterbalanced across children.

Finally, children completed the Baby Stroop Task (Hughes and Ensor, 2005). Children participated in a “silly game” in which they pointed to a large spoon when the examiner said “Baby” and a small spoon when the examiner said “Mummy.” Children completed 6 trials (with feedback) and passed (earning a score of “1”) if they performed correctly on 4 or more trials (*n* = 43); children who performed correctly on fewer than 4 trials received a score of 0 (*n* = 124).

We created an EF score by summing together the number of tasks each child passed, such that children could receive a score between 0 and 4 (Hughes et al., 2020).

##### EF at 36 months

Children first completed the Spin the Pots task, a different multi-location search task designed to test children’s working memory (Hughes and Ensor, 2005). Six raisins were hidden beneath eight paper cups on a lazy Susan tray (two cups were empty). Each of the eight cups was a different color. After raisins were hidden, the entire display was covered by a cloth and the tray was rotated 180 degrees. The cloth was then removed and children were instructed to “show me which cup you want to open”. If the child chose a cup with a raisin in it, the child was told “Good job! Well done. You got a raisin! Let’s put your raisin in here for later” after which the raisin was visibly and obviously removed from the cup and placed in an envelope. If the child chose a cup with no raisin, the child was told “Oh no. There’s no raisin there. Let’s try another.” Testing was discontinued either when the child had received all six raisins or when 12 trials had been administered. Children received a score of “1” (*n* = 63) if they found five of six raisins, “2” (*n* = 73) if they found all six raisins, and a score of “0” (*n* = 29) if they found fewer than five raisins.

Children then completed a version of the Dimensional Change Card Sorting task (Zelazo, 2006), wherein they were counterbalanced across a color-first or shape-first condition. Children were first familiarized with the cards (a blue rabbit and a red boat), then saw one example trial and received one training trial with feedback. In the “color game” children were asked to place all the cards of a given color in the appropriate pile. Children received six test trials with no feedback in a standardized order with a reminder of the rule at the beginning of each (“Remember, if it’s blue it goes here and if it’s red it goes there. Here’s a red/blue one. Where does it go?”). In the shape game, children were asked to place all the cards of a given shape in the appropriate pile. Again, children received six test trials with no feedback in a standardized order with a reminder of the rule at the beginning of each (“Remember, if it’s a rabbit it goes here, and if it’s a boat it goes there. Here’s a rabbit/boat, where does it go?”). If a child was incorrect on four or more trials of the first test condition, testing was discontinued; if the child was correct on three or more trials, they moved to other game. Children passed a phase if they performed correctly on 4 or more trials, such that they could receive a score of “0” (*n* = 17), “1” (*n* = 96), or “2” (*n* = 45).

Next, children completed the *Baby Stroop Task* described above (Hughes and Ensor, 2005). In the version administered when children were 36 months, children received a total of 16 test trials. To avoid fatigue, half the trials were completed with spoons as at 24 month, and half were completed with cups. Again, children played a “silly game” in which they pointed to a large spoon/cup when the examiner said “Baby” and a small spoon/cup when the examiner said “Mummy.” Assessment was discontinued if children were incorrect on three trials in either the spoon or cup condition. For the purpose of analysis, the two different conditions (spoons and cups) were treated as separate tasks and children passed each trial (and thus received a score of “1” if they performed correctly on 6 or more trials, such that they could receive a score of “0” (*n* = 37), “1” (*n* = 36), or “2” (*n* = 72).

Finally, children completed a self-ordered pointing task (Cragg and Nation, 2007; Devine et al., 2016). Children were shown a flipbook with an increasing number of pictures of single-syllable objects (ranging from 2 to 6) in 1 of 16 locations on a page. For example, the first page depicted two objects (e.g., doll and belt) and the next page had the same two objects in two different locations. Children were required to point to a new picture on each page and were told to not select the same picture twice. The task began with two practice trials with experimenter feedback. All children completed two test trials for each number of objects (3, 4, 5, and 6 objects) for a total of eight test trials. A span score was assigned based on the highest number of objects for which the child made zero errors on at least one of the two test trials. Children received a score of “0” if their span score was below 3 (*n* = 47), “1” if their span score was 3 or 4 (*n* = 83), and a score of “2” if their span score was 5 or 6 (*n* = 24).

An EF score was again created by summing together the number of tasks each child passed, such that children could receive a score between 0 and 8. In addition to reducing the number of variables in our models, we opted for a single aggregate score for EF because these scores exhibit greater stability over time than individual task scores (Miller and Marcovitch, 2015). Both EF aggregates were adequately reliable; reliability coefficient (i.e., ordinal alpha based on tetrachoric correlations) was modest at both 14 months (α = 0.58) and 24 months (α = 0.49). These results were consistent with the modest EF task correlations in this age range (Kochanska and Knaack, 2003; Miller and Marcovitch, 2015; Johansson et al., 2016).

### Covariates

A series of covariates was included in analyses to ensure inferences are not due to level of understanding or other child characteristics. In addition to EF at T1, covariates included child age at each time point (thereby also effectively controlling for length of time between testing time points), child sex, and receptive vocabulary at both T1 and T2. In addition, covariates describing parent age at time of child birth, parents’ subjective social status (“This ladder represents where people stand in society. Where would you be on this ladder?” On a range from “1” = “The worst off people are at the bottom of the ladder–these people have the least education and money and the worst jobs” to “10” = “The best off people are at the top of the ladder–these people have the most education and money and the best jobs.”) and whether or not parent had received higher than a bachelor’s degree were included in analyses. Receptive vocabulary was measured using the receptive vocabulary subtest of the Wechsler Preschool and Primary Scales of Intelligence. Children were asked to point to one of four images that corresponded to word read aloud by the experimenter; participants completed up to 38 trials of increasing difficulty. Testing was discontinued after children were incorrect on 5 consecutive trials. The total score was used to provide an index for verbal ability.

## Results

### Descriptive Statistics

Descriptive statistics are displayed in Table 1, and bivariate correlations among all variables are displayed in Table 2. Most children engaged in screen time at both 24- and 36-month time points, and screen time increased as children got older. Individual differences were stable across timepoints, *r*(147) = 0.579, *p* < 0.001. Mean screen time usage increased significantly from T1 (*M*_T__1_ = 86.86, *SD* = 64.99) to T2 (*M*_T__2_ = 116.18, *SD* = 52.53), paired-sample *t*(148) = 6.53, *p* < 0.001. There were no gender differences in screen use at either timepoint [T1: *t*(177) = −0.80, *p* = 0.423; T2: *t*(147) = 0.50, *p* = 0.617]. Individual differences in EF were modestly stable from 24- to 36-months, *r*_s_(168) = 0.15, *p* = 0.050, consistent with prior findings on stability in EF (Carlson et al., 2004; Miller and Marcovitch, 2015; Hughes et al., 2020).

**TABLE 1:** Descriptive statistics and frequencies for all study variables.

	*N*	Min.	Max.	Mean	SD
EF 24 months	179	0	4	2.15	1.05
EF 36 months	163	0	8	4.14	1.79
Screen time 24 months	179	0	384.64	84.95	61.79
Screen time 36 months	149	34	372.5	116.18	52.53
WPPSI 24 months	140	0	23	10.34	5.02
WPPSI 36 months	160	0	29	18.12	5.67
Age 24 months	179	20.34	26.97	24.29	0.85
Age 36 months	163	34.79	40.15	36.73	1.06
Mother age at child birth	177	25.10	43.15	32.68	3.68
Father age at child birth	174	23.76	49.63	34.17	4.45
Mother subjective social status	179	3.67	10	7.37	1.18
Father subjective social status	179	4.33	10	7.34	1.10
Child gender	179				
Male	100				
Female	79				
Mother more than bachelor degree	177				
Yes	75				
No	102				
Father more than bachelor degree	176				
Yes	68				
No	108				

**TABLE 2:** Bivariate Pearson correlations among study variables.

		**1**	**2**	**3**	**4**	**5**	**6**	**7**	**8**	**9**	**10**	**11**	**12**	**13**	**14**	**15**
1	Executive Function 24mo	−														
2	Executive Function 36mo	0.15	−													
3	Screen Time 24mo	–0.05	–0.12	−												
4	Screen Time 36mo	0.03	0.06	0.56^∗∗∗^	−											
5	WPPSI 24mo	0.15	0.27^∗∗^	0.03	0.07	−										
6	WPPSI 36mo	0.10	0.29^∗∗∗^	–0.10	–0.07	0.28^∗∗^	−									
7	Child Age 24mo	0.16^∗^	0.03	0.09	–0.11	0.24^∗∗^	0.04	−								
8	Child Age 36mo	0.06	0.08	–0.01	–0.09	0.15	0.10	0.49^∗∗∗^	−							
9	Child Female	0.16^∗^	0.20^∗^	0.06	–0.04	0.04	0.14	–0.11	–0.15	−						
10	Mother >Bach. Deg.	0.08	–0.04	–0.24^∗∗^	–0.31^∗∗∗^	0.05	0.03	–0.02	0.05	0.07	−					
11	Father >Bach. Deg.	0.07	0.07	–0.34^∗∗∗^	–0.32^∗∗∗^	0.12	0.03	0.02	–0.01	–0.03	0.29^∗∗∗^	−				
12	Mother Age at Child Birth	0.00	–0.04	–0.01	−0.16^∗^	0.08	0.05	0.01	–0.07	0.01	0.18^∗^	0.08	−			
13	Father Age at Child Birth	–0.06	–0.06	–0.05	–0.23^∗∗^	0.03	0.06	0.02	–0.07	0.02	0.10	0.11	0.67^∗∗∗^	−		
14	Mother Subj. Social Status	0.03	0.06	−0.19^∗^	–0.25^∗∗^	–0.05	0.08	–0.03	–0.02	0.10	0.26^∗∗∗^	0.20^∗∗^	0.15^∗^	0.14	−	
15	Father Subj. Social Status	0.07	0.06	–0.24^∗∗^	−0.18^∗^	–0.12	0.14	–0.11	0.05	–0.02	0.17^∗^	0.34^∗∗∗^	0.00	–0.01	0.39^∗∗∗^	−

*^∗∗∗^*p*< 0.001, ^∗∗^*p*< 0.01, ^∗^*p*< 0.05; > Bach. Deg.—More than Bachelor’s Degree; Subj. Social Status—Subjective Social Status; WPPSI—Wechsler Preschool and Primary Scales of Intelligence Receptive Vocabulary Score.*

### Regression Analysis

We next ran an OLS regression model to test the hypothesis that screen time is negatively associated with children’s EF at 36 months of age. We tested the association of both concurrent and prior screen use; results are shown in Table 3. Concurrent screen time was not significantly associated with children’s EF such that there appeared to be no direct relation between screen use and EF when children were 36 months of age (*p* = 0.069); however, screen time from T1 (when children were 24 months) was negatively associated with children’s EF at 36 months, suggesting longitudinal implications for screen use. Screen time at T1 was negatively associated with EF at T2, β = −0.20, *p* = 0.035, such that an increase in one standard deviation of screen time (64.99 min) was associated with nearly a quarter standard deviation (0.48 of eight possible points) in EF.

**TABLE 3:** Linear regression predicting EF at 36 months.

	β	*SE*	*p-value*
Executive function 24 months	0.05	0.08	0.500
Screen time 24 months	–0.20	0.09	0.032
Screen time 36 months	0.18	0.10	0.071
WPPSI 24 months	0.15	0.09	0.115
WPPSI 36 months	0.20	0.08	0.018
Child age 24 months	0.00	0.09	0.961
Child age 36 months	0.09	0.09	0.303
Child female	0.17	0.08	0.022
Mother more than bachelor degree	–0.09	0.08	0.243
Father more than bachelor degree	0.06	0.08	0.502
Mother age at child birth	0.01	0.10	0.959
Father age at child birth	–0.04	0.10	0.678
Mother subjective social status	0.05	0.08	0.560
Father subjective social status	0.02	0.08	0.855

*WPPSI, Wechsler preschool and primary scales of intelligence receptive vocabulary score.*

To better understand the direction of these associations, we tested an alternative hypothesis whereby children with worse EF spend more time engaged with digital media. To test this alternate direction of association, a sensitivity test was run wherein EF at T1 and T2, as well as screen time at T1, were used to predict screen time at T2. The same covariates (i.e., receptive vocabulary, gender, and age) were included. The only significant predictor of screen use at T2 was screen use at T1: β = 0.48, *p* < 0.001.

## Discussion

As expected, the current study found that, controlling for receptive vocabulary, gender, age, and prior EF, there was a linear relation between screen time at 24 months and EF 1 year later, such that increased screen time was associated with worse EF. Contrary to expectations, however, concurrent screen time was not associated with EF when children were 36 months of age.

The longitudinal findings of the current study are concordant with prior longitudinal findings (e.g., Barr et al., 2010; McHarg et al., 2020). This association may be due to increased screen use replacing activities that are important for cognitive development, such as playing with manipulatives and engaging in imaginative play. When these activities are replaced by screen time, executive function development may be permanently and negatively impacted. Indeed, the current findings suggest digital media use is implicated in EF development longitudinally in a “trait” fashion, rather than simply in a short-term “state” effect which might be suggested by concurrent relations. This impact is critical – if early television exposure is impacting executive function in a long-term manner, seemingly innocuous media exposure may have detrimental effects on academic achievement, socioemotional learning, and more. Thus, the current findings suggest that the WHO and AAP guidelines are justifiable.

Alternatively, children who are more inclined to view television more often, or parents who may be more likely to use television as everyday entertainment for their children, may have lower executive functioning due to genetic or other environmental factors. Indeed, Cliff et al. (2018) found that more screen time at 2-years was associated with lower self-regulation at 4-years. This association was strongest for children with highly educated parents, and may be child-driven (e.g., parents using more screen time to cope with children with self-regulation difficulties). Future research should investigate the potential mediating factor of parental EF on these associations.

Notably, concurrent screen time and EF were unrelated. This may be due to children viewing more child-directed content as they get older, as opposed to most television viewing in infancy and toddlerhood consisting of exposure to adult-directed content. Indeed, one study by Barr et al. (2010) found negative associations between specific aspects of screen exposure and EF. Using a longitudinal study, the authors reported that while exposure to adult-directed content was associated with lower EF, exposure to child-directed screen content was unrelated to EF. It is well-established that parent-child interactions are impaired when adult-directed content is on in the background (Kirkorian et al., 2009). It could be that children who are exposed to more adult-directed content miss out on important interactions with parents, or other exploration that is important for EF development. In contrast, child-directed content often includes information that is helpful for EF development, or at least incorporates enough of these things that development is not impaired by screen viewing. Importantly, however, it could be that longitudinal associations are significant because the negative impacts on development might be cumulative rather than immediate; concurrent associations might not be as easily detected as negative impacts of digital media use over time. The differences between the concurrent and longitudinal effects are important examples of the complex relation between executive function and screen time longitudinally, and highlight the need for caution in interpretation.

Another important consideration is the varied elements of EF. Some prior research has suggested that screen time may influence different executive functions differently. For example, Huber et al. (2018) found in a sample of 96 2- and 3-year-old children, those who engaged in tablet play were more likely to delay gratification than children who watched a cartoon; working memory increased after tablet play. Similarly, McHarg et al. (2020) found that screen exposure at 4-months was associated with decreased inhibition at 14-months, but was not significantly associated with working memory or set-shifting. However, due to the strong association between different elements of EF and the different test batteries at different time points, a composite score of EF was used in the current study. Future work should develop robust task batteries that include several measures of each element of EF and investigate these longitudinally.

### Strengths and Limitations

Strengths of the current work include the robust measurement of both EF and screen exposure across time-points in an age group that is understudied with respect to longitudinal trends in digital media usage. In addition, our regression analyses included vocabulary to ensure effects were specific to EF.

Three key limitations also deserve note. First, while this longitudinal investigation offers a unique opportunity to test and understand long-run implications of early media use on *gains* in EF over time, the study lacked the experimental design needed to infer causality. In particular, another unmeasured variable may account for the development of both children’s EF and screen viewing. Second, to minimize participant burden, reflective surveys rather than a screen time diary or device monitoring system were used to assess digital media use. Though the survey included the opportunity to note which devices children were exposed to over time, it did not record specific information about what children were doing on each device, such as playing an interactive game or viewing a film. In addition, the current study did not account for background television and context of viewing (e.g., while eating, co-viewing with a parent, viewing to calm a child down vs. to allow a parent to accomplish a task, etc.). Further work is needed to identify fine-grained associations between digital media and EF development. Third, to avoid lengthy assessments we included just 3–4 EF tasks at each time-point and so were limited in our ability to investigate individual EF components in detail. Future work should establish research designs that allow for longitudinal investigations that can test the factor structure of EF within the dataset and, if appropriate, test the predictive value of screen time for different aspects of EF.

### Conclusion

Overall, the current longitudinal findings strengthen a growing body of literature on associations between screen-based digital media exposure and EF in toddlerhood. The findings discussed here highlight the potentially detrimental impacts of increased digital media exposure in toddlerhood on cognitive development. EF is a complex construct, and is influenced by several environmental and heritable factors, some of which cannot be controlled. However, though digital media exposure is ubiquitous in a modern childhood, parents, educators, and caretakers should exercise caution when exposing young children to large amounts of screen time.

## Data Availability Statement

The raw data supporting the conclusions of this article will be made available by the authors, without undue reservation.

## Ethics Statement

The studies involving human participants were reviewed and approved by the University of Cambridge Ethics Committee and National Health Service (NHS, United Kingdom) Research Ethics Committee approved the study protocol (REF: 14/LO/1113). Written informed consent to participate in this study was provided by the participants’ legal guardian/next of kin.

## Author Contributions

GM, RD, and CH were involved in data collection. All authors were involved in study design, data analysis, and manuscript preparation.

## Conflict of Interest

The authors declare that the research was conducted in the absence of any commercial or financial relationships that could be construed as a potential conflict of interest.

## References

[B1] Anderson D. R. Subrahmanyam K. (2017). Digital screen media and cognitive development. *Pediatrics* 140(Suppl. 2) S57–S61. 10.1542/peds.2016-1758C 29093033

[B2] Barr R. Lauricella A. Zack E. Calvert S. L. (2010). Infant and early childhood exposure to adult-directed and child-directed television programming: relations with cognitive skills at age four. *Merrill Palmer Q.* 56 21–48. 10.1353/mpq.0.0038

[B3] Blair C. (2010). Stress and the development of self-regulation in context. *Child Dev. Perspect.* 4 181–188. 10.1111/j.1750-8606.2010.00145.x 21779305 PMC3138186

[B4] Blankenship T. L. Slough M. A. Calkins S. D. Deater-Deckard K. Kim-Spoon J. Bell M. A. (2019). Attention and executive functioning in infancy: links to childhood executive funciton and reading achievement. *Dev. Sci.* 22:e12824. 10.1111/desc.12824 30828908 PMC6722030

[B5] Carlson S. M. Mandell D. J. Williams L. (2004). Executive function and theory of mind: stability and prediction from ages 2 to 3. *Dev. Psychol.* 40, 1105–1122. 10.1037/0012-1649.40.6.1105 15535760

[B6] Chassiakos Y. L. R. Radesky J. Christakis D. Moreno M. A. Cross C. (2016). Children and adolescents and digital media. *Pediatrics* 138:e20162593. 10.1542/peds.2016-2593 27940795

[B7] Cheung C. H. M. Bedford R. Saez de Urabain I. R. Karmiloff-Smith A. Smith T. J. (2017). Daily touchscreen use in infants and toddlers is associated with reduced sleep and delayed sleep onset. *Sci. Rep.* 7:46104. 10.1038/srep46104 28406474 PMC5390665

[B8] Christakis D. A. Gilkerson J. Richards J. A. Zimmerman F. J. Garrison M. M. Xu D. (2009). Audible television and decreased adult words, infant vocalizations, and conversational turns: a population-based study. *Arch. Pediatr. Adolesc. Med.* 163 554–558. 10.1001/archpediatrics.2009.61 19487612

[B9] Cliff D. P. Howard S. J. Radesky J. S. McNeill J. Vella S. A. (2018). Early childhood media exposure and self-regulation: bidirectional longitudinal associations. *Acad. Pediatr.* 18 813–819. 10.1016/j.acap.2018.04.012 29704999

[B10] Cragg L. Nation K. (2007). Self-ordered pointing as a test of working memory in typically developing children. *Memory* 15 526–535. 10.1080/09658210701390750 17613795

[B11] Devine R. T. Bignardi G. Hughes C. (2016). Executive function mediates the relations between parental behaviors and children’s early academic ability. *Front. Psychol.* 7:1902. 10.3389/fpsyg.2016.01902 28018253 PMC5156724

[B12] Devine R. T. Hughes C. (2014). Relations between false belief understanding and executive function in early childhood: a meta-analysis. *Child Dev.* 85 1777–1794. 10.1111/cdev.12237 24605760

[B13] Devine R. T. Ribner A. Hughes C. (2019). Measuring and predicting individual differences in executive functions at 14 months: a longitudinal study. *Child Dev.* 90:e618–e636. 10.1111/cdev.13217 30663776 PMC6849706

[B14] Diamond A. (2013). Executive functions. *Annu. Rev. Psychol.* 64 135–168. 23020641 10.1146/annurev-psych-113011-143750 PMC4084861

[B15] Drotner K. (2013). “The co-construction of media and childhood,” in *The Routledge International Handbook of Children, Adolescents and Media*, ed. Lemish D. (Abingdon: Routledge), 15–22.

[B16] Duncan R. J. Schmitt S. A. Vandell D. L. (2019). Additive and synergistic relations of early mother–child and caregiver–child interactions for predicting later achievement. *Dev. Psychol.* 55 2522–2533. 10.1037/dev0000824 31535893 PMC6861641

[B17] Fay-Stammbach T. Hawes D. J. Meredith P. (2014). Parenting influences on executive function in ealry childhood: a review. *Child Dev. Perspect.* 8 258–264. 10.1111/cdep.12095

[B18] Fiske A. Holmboe K. (2019). Neural substrates of early executive function development. *Dev. Rev.* 52 42–62. 10.1016/j.dr.2019.100866 31417205 PMC6686207

[B19] Friedman N. P. Miyake A. Altamirano L. J. Corley R. P. Young S. E. Rhea S. A. (2016). Stability and change in executive function abilities from late adolescence to early adulthood: a longitudinal twin study. *Dev. Psychol.* 52 326–340. 10.1037/dev0000075 26619323 PMC4821683

[B20] Hendry A. Jones E. J. H. Charman T. (2016). Executive function in the first three years of life: precursors, predictors and patterns. *Dev. Rev.* 42 1–33. 10.1016/j.dr.2016.06.005

[B21] Hipp D. Gerhardstein P. Zimmermann L. Moser A. Taylor G. Barr R. (2017). “The dimensional divide: learning from TV and touchscreens during early childhood,” in *Media Exposure During Infancy and Early Childhood.* eds Barr R. Linebarger D. N. (Cham: Springer), 33–54. 10.1007/978-3-319-45102-2_3

[B22] Hisler G. Twenge J. M. Krizan Z. (2020). Associations between screen time and short sleep duration among adolescents varies by media type: evidence from a cohort study. *Sleep Med.* 66 92–102. 10.1016/j.sleep.2019.08.007 31838456

[B23] Holmboe K. Bonneville-Roussy A. Csibra G. Johnson M. H. (2018). Longitudinal development of attention and inhibitory control during the first year of life. *Dev. Sci.* 21:e12690. 10.1111/desc.12690 29911347

[B24] Huber B. Yeates M. Meyer D. Fleckhammer L. Kaufman J. (2018). The effects of screen media content on young children’s executive functioning. *J. Exp. Child Psychol.* 170 72–85. 10.1016/j.jecp.2018.01.006 29448235

[B25] Hughes C. Devine R. T. (2019). For better or for worse? Positive and negative parental influences on young children’s executive function. *Child Dev.* 90 593–609. 10.1111/cdev.12915 28800148

[B26] Hughes C. Devine R. T. Mesman J. Blair C. (2020). Understanding the terrible twos: a longitudinal investigation of the impact of early executive function and parent-child interactions. *Dev. Sci.* e12979. 10.1111/desc.12979 [Epub ahead of print]. 32353921

[B27] Hughes C. Ensor R. (2005). Executive function and theory of mind in 2 year olds: a family affair? *Dev. Neuropsychol.* 28 645–668. 10.1207/s15326942dn2802_5 16144431

[B28] Hughes C. Ensor R. Wilson A. Graham A. (2009). Tracking executive function across the transition to school: a latent variable approach. *Dev. Neuropsychol.* 35 20–36. 10.1080/87565640903325691 20390590

[B29] Hughes C. Roman G. Hart M. J. Ensor R. (2013). Does maternal depression predict young children’s executive function?–a 4-year longitudinal study. *J. Child Psychol. Psychiatry* 54 169–177. 10.1111/jcpp.12014 23171379

[B30] Johansson M. Marciszko C. Brocki K. Bohlin G. (2016). Individual differences in early executive functions: a longitudinal study from 12 to 36 months. *Infant Child Dev.* 25, 533–549. 10.1002/icd.1952

[B31] Kirkorian H. Pempek T. Choi K. (2017). “The role of online processing in young children’s learning from interactive and noninteractive digital media,” in *Media Exposure During Infancy and Early Childhood*, eds Barr R. Linebarger D. N. (Cham: Springer), 65–89. 10.1007/978-3-319-45102-2_5

[B32] Kirkorian H. L. Pempek T. A. Murphy L. A. Schmidt M. E. Anderson D. R. (2009). The impact of background television on parent-child interaction. *Child Dev.* 80 1350–1359. 10.1111/j.1467-8624.2009.01337.x 19765004

[B33] Kochanska G. Knaack A. (2003). Effortful control as a personality characteristic of young children: antecedents, correlates, and consequences. *J. Pers.* 71, 1087–1112. 10.1111/1467-6494.7106008 14633059

[B34] Lerner M. D. Lonigan C. J. (2014). Executive function among preschool children: unitary versus distinct abilities. *J. Psychopathol. Behav. Assess.* 36 626–639. 10.1007/s10862-014-9424-3 25642020 PMC4306461

[B35] Lillard A. S. Drell M. B. Richey E. M. Boguszewski K. Smith E. D. (2015). Further examination of the immediate impact of television on children’s executive function. *Dev. Psychol.* 51, 792–805. 10.1037/a0039097 25822897

[B36] Lillard A. S. Peterson J. (2011). The immediate impact of different types of television on young children’s executive function. *Pediatrics* 128 644–649. 10.1542/peds.2010-1919 21911349 PMC9923845

[B37] Madigan S. Browne D. Racine N. Mori C. Tough S. (2019). Association between screen time and children’s performance on a developmental screening test. *JAMA Pediat*. 10.1001/jamapediatrics.2018.5056 30688984 PMC6439882

[B38] Magee C. A. Blunden S. (2020). Sleep timing during adolescence: a latent transition analysis approach. *Behav. Sleep Med.* 18 131–146. 10.1080/15402002.2018.1546180 30472878

[B39] McHarg G. Ribner A. D. Devine R. T. Hughes C. NewFAMS Study Team (2020). Infant screen exposure links to toddlers’ inhibition, but not other EF constructs: a propensity score study. *Infancy* 25 205–222. 10.1111/infa.12325 32749042

[B40] Mendelsohn A. L. Berkule S. B. Tomopoulos S. Tamis-LeMonda C. S. Huberman H. S. Alvir J. (2008). Infant television and video exposure associated with limited parent-child verbal interactions in low socioeconomic status households. *Arch. Pediatr. Adolesc. Med.* 162 411–417. 10.1001/archpedi.162.5.411 18458186 PMC3081686

[B41] Miller S. E. Marcovitch S. (2015). Examining executive function in the second year of life: coherence, stability, and relations to joint attention and language. *Dev. Psychol.* 51 101–114. 10.1037/a0038359 25546598

[B42] Mulder H. Verhagen J. Van der Ven S. H. Slot P. L. Leseman P. P. (2017). Early executive function at age two predicts emergent mathematics and literacy at age five. *Front. Psychol.* 8:1706. 10.3389/fpsyg.2017.01706 29075209 PMC5643463

[B43] Nathanson A. I. Aladé F. Sharp M. L. Rasmussen E. E. Christy K. (2014). The relation between television exposure and executive function among preschoolers. *Dev. Psychol.* 50 1497–1506. 10.1037/a0035714 24447117

[B44] Ribner A. Barr R. F. Nichols D. L. (2020). Media use and school readiness skills: indirect effects of self-regulation. *Pediatr. Res.* 10.1038/s41390-020-1004-5 32521541

[B45] Ribner A. McHarg G. (2019). Why won’t she sleep? Screen expsoure and sleep patterns in young infants. *Infant Behav. Dev.* 57:101334. 10.1016/j.infbeh.2019.101334 31229915

[B46] Robinson T. N. Banda J. A. Hale L. Lu A. S. Fleming-Milici F. Calvert S. L. (2017). Screen media exposure and obesity in children and adolescents. *Pediatrics* 140 S97–S101. 10.1542/peds.2016-1758K 29093041 PMC5769928

[B47] Scionti N. Cavallero M. Zogmaister C. Marzocchi G. M. (2020). Is cognitive training effective for improving executive functions in preschoolers? A systematic review and meta-analysis. *Front. Psychol.* 10:2812. 10.3389/fpsyg.2019.02812 31998168 PMC6965160

[B48] Short S. J. Willoughby M. T. Camerota M. Stephens R. L. Steiner R. J. Styner M. (2019). Individual differences in neonatal white matter are associated with executive function at 3 years of age. *Brain Struct. Funct.* 224 3159–3169. 10.1007/s00429-019-01955-0 31520254

[B49] Vernon-Feagans L. Willoughby M. Garrett-Peters P. (2016). Predictors of behavioral regulation in kindergarten: household chaos, parenting, and early executive functions. *Dev. Psychol.* 52 430–441. 10.1037/dev0000087 26751500 PMC4760868

[B50] Viner R. Davie M. Firth A. (2019). *The Health Impacts of Screen Time: A Guide for Clinicians and Parents.* Available online at: https://www.rcpch.ac.uk/sites/default/files/2018-12/rcpch_screen_time_guide_-_final.pdf (accessed January 30, 2019).

[B51] Willoughby M. T. Blair C. B. Wirth R. J. Greenberg M. (2010). The measurement of executive function at age 3 years: psychometric properties and criterion validity of a new battery of tasks. *Psychol. Assess.* 22 306–317. 10.1037/a0018708 20528058

[B52] Willoughby M. T. Magnus B. Vernon-Feagans L. Blair C. B. Family Life and Project Investigators. (2017). Developmental delays in executive function from 3 to 5 years of age predict kindergarten academic readiness. *J. Learn. Disabil.* 50 359–372. 10.1177/0022219415619754 26755570 PMC5266699

[B53] Willoughby M. T. Wirth R. J. Blair C. B. (2012). Executive function in early childhood: longitudinal measurement invariance and developmental change. *Psychol. Assess.* 24 418–431. 10.1037/a0025779 22023561 PMC3495312

[B54] World Health Organization (2019). *Guidelines on Physical Activity, Sedentary Behaviour and Sleep for Children under 5 Years of Age.* Available online at: https://apps.who.int/iris/handle/10665/311664 (accessed April 2, 2019). 31091057

[B55] Zelazo P. D. (2006). The dimensional change card sort (DCCS): a method of assessing executive function in children. *Nat. Protoc.* 1 297–301. 10.1038/nprot.2006.46 17406248

